# Matrix Rigidity Induces Osteolytic Gene Expression of Metastatic Breast Cancer Cells

**DOI:** 10.1371/journal.pone.0015451

**Published:** 2010-11-15

**Authors:** Nazanin S. Ruppender, Alyssa R. Merkel, T. John Martin, Gregory R. Mundy, Julie A. Sterling, Scott A. Guelcher

**Affiliations:** 1 Department of Chemical and Biomolecular Engineering, Vanderbilt University, Nashville, Tennessee, United States of America; 2 Center for Bone Biology, Vanderbilt University Medical Center, Nashville, Tennessee, United States of America; 3 Division of Clinical Pharmacology, Department of Medicine, Vanderbilt University Medical Center, Nashville, Tennessee, United States of America; 4 Veterans' Affairs Tennessee Valley Healthcare System, Nashville, Tennessee, United States of America; 5 Department of Cancer Biology, Vanderbilt University Medical Center, Nashville, Tennessee, United States of America; 6 Saint Vincent's Institute, Melbourne, Victoria, Australia; Health Canada, Canada

## Abstract

Nearly 70% of breast cancer patients with advanced disease will develop bone metastases. Once established in bone, tumor cells produce factors that cause changes in normal bone remodeling, such as parathyroid hormone-related protein (PTHrP). While enhanced expression of PTHrP is known to stimulate osteoclasts to resorb bone, the environmental factors driving tumor cells to express PTHrP in the early stages of development of metastatic bone disease are unknown. In this study, we have shown that tumor cells known to metastasize to bone respond to 2D substrates with rigidities comparable to that of the bone microenvironment by increasing expression and production of PTHrP. The cellular response is regulated by Rho-dependent actomyosin contractility mediated by TGF-ß signaling. Inhibition of Rho-associated kinase (ROCK) using both pharmacological and genetic approaches decreased PTHrP expression. Furthermore, cells expressing a dominant negative form of the TGF-ß receptor did not respond to substrate rigidity, and inhibition of ROCK decreased PTHrP expression induced by exogenous TGF-ß. These observations suggest a role for the differential rigidity of the mineralized bone microenvironment in early stages of tumor-induced osteolysis, which is especially important in metastatic cancer since many cancers (such as those of the breast and lung) preferentially metastasize to bone.

## Introduction

Nearly 70% of breast cancer patients with advanced disease will develop bone metastases that are commonly associated with pain, hypercalcemia, and pathologic fracture [1]. Once established in bone, tumor cells begin to produce factors that cause changes in normal bone remodeling. The best-described example is the expression of parathyroid hormone-related protein (PTHrP), which is expressed at higher levels in bone metastases from breast cancers than it is in isolated primary tumors or soft tissue metastases [2], [3]. In the bone microenvironment, enhanced expression of PTHrP stimulates osteoclasts to resorb bone [4]. As the bone is resorbed, the release of transforming growth factor beta (TGF-ß) from the bone matrix contributes to further increase PTHrP expression [5]. Thus, while TGF-ß released from the bone matrix sustains the “vicious cycle” of bone resorption in the later stages of bone disease, the environmental factors driving tumor cells to express PTHrP in the early stages of development of metastatic bone disease prior to bone resorption are unknown.

In addition to its osteolyic function in metastatic bone disease, PTHrP also performs a number of normal physiological functions, including the regulation of smooth muscle tone. Mechanically transduced signals have been shown to regulate PTHrP expression and secretion in a variety of smooth muscle beds [6]. Mechanical distension of the abdominal aorta [7], the uterus, and the bladder [8] in rats increased PTHrP expression by a factor of two or more. Because of the dramatically (∼10^6^) higher rigidity of mineralized bone tissue compared to breast tissue, tumor cells are likely to generate higher cytoskeleton-dependent forces in the bone microenvironment. Therefore, we hypothesized that the differential rigidity of the bone microenvironment might influence PTHrP expression by tumor cells through mechanically transduced signals.

Previous studies have shown that matrix rigidity regulates invasiveness at the primary site [9], [10]. When cells encounter a mechanically rigid matrix, integrins become activated, which stimulates RhoGTPase-dependent actomyosin contractility. However, while normal cells tune their contractility in response to matrix rigidity, tumor cells exhibit altered tensional homeostasis, as evidenced by their higher contractility and spreading relative to non-malignant mammary epithelial cells on compliant matrices [11]. Inhibition of RhoGTPase signaling in tumor cells by treating with Rho-associated kinase (ROCK) inhibitors reduces tumor cell contractility and spreading [11]. Additionally, ROCK expression is higher in metastatic human mammary tumors relative to non-metastatic tumors, and inhibition of ROCK signaling decreases cell proliferation *in vitro* and metastasis to bone *in vivo* [12].

To test our hypothesis that cytoskeleton-dependent forces regulate PTHrP expression in tumor cells, we designed a 2D tumor cell mono-culture system. Previous studies investigating the effects of matrix rigidity on cell migration, differentiation, and invasion have utilized *in vitro* cell culture on 2D model substrates, such as Matrigel™ [13], [14], crosslinked gelatin [15], and synthetic hydrogels [16], [17]. However, the applicability of these substrates to the bone microenvironment is limited, due to the inability to achieve a sufficiently high elastic modulus that is relevant to mineralized bone tissue. In this study, we prepared polyacrylamide (PAA) hydrogels as a model for breast tissue and poly(ester urethane) films [18], [19] as a model for tissues ranging from the basement membrane to bone. We measured changes in gene expression by metastatic tumor cells in response to the rigidity of the substrate. In addition, we investigated the effects of ROCK and TGF-ß inhibition and stimulation using pharmacological agents and genetically modified cells to identify the signaling pathways through which rigidity regulates gene expression.

## Materials and Methods

### Materials

#### Materials synthesis

**Figure 1:**
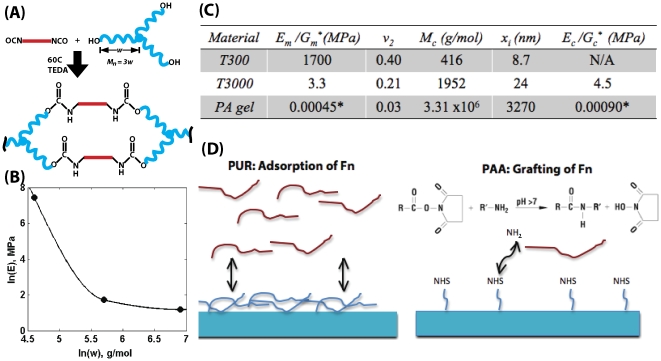
Schematic of materials synthesis and characterization. (A) Synthesis of PUR networks from an LDI quasi-prepolymer (red) and a poly(ε-caprolactone-co-glycolide) triol with molecular weight M_n_ = 3*w*, where *w* is the equivalent weight (g eq^−1^). (B) Experimental values of the elastic modulus *E* of PUR networks as a function of equivalent weight *w*. (C) Physical characteristics of polymer networks as calculated from rubber elasticity theory. E_m_ and G_m_ represents measured elastic and shear moduli, 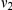 is the fraction of polymer in the swollen mass, M_c_ is the calculated molecular weight between cross-links and E_c_ and G_c_ are the calculated elastic and shear moduli. (D) Schemes for providing a uniform surface concentration of fibronectin (Fn) for polyurethane networks and polyacrylamide gels.

Methyl 2,6-diisocyanatohexane (lysine methyl ester diisocyanate, LDI) was purchased from Kyowa Hakko USA (New York, NY). The structures of these polyisocyanates are shown in Figure 1. Coscat 83, an organobismuth urethane catalyst, was supplied by ChasChem, Inc. (Rutherford, NJ). Stannous octoate, glycerol, poly(6-caprolactone) triol (300 Da), and 6-caprolactone were purchased from Aldrich (St. Louis, MO), and glycolide was purchased from Polysciences (Warrington, PA). Glycerol was dried at 10 mm Hg for 3 hours at 80°C and caprolactone was dried over anhydrous magnesium sulfate prior to use. All other materials were used as received. Two-component cast poly(ester urethane)s were mixed using a Hauschild SpeedMixer™ DAC 150 FVZ-K (FlackTek Inc., Landrum, SC).

#### Cell culture

Dulbecco's modification of Eagle's medium (DMEM) and McCoy's 5A were purchased from Invitrogen (Carlsbad, CA). Fetal bovine serum (FBS) was purchased from Hyclone Laboratories (Logan, UT). Penicillin, streptomycin, L-glutamine, trypsin, sodium pyruvate, essential and non-essential amino acids were all acquired from Mediatech (Manassas, VA). MDA-MB-231, RWGT2 and MCF-7 cells were purchased from ATCC (Manassas, VA). MDA-MB-231 and RWGT2 cells were then selected for ability to metastasize to bone [20].

#### Antibodies, Primers and Reagents

The RNeasy™ mini kit was purchased from Qiagen (Valencia, CA). Superscript III cDNA synthesis kits were purchased from Invitrogen (Carlsbad, CA). qPCR primers for PTHrP, Gli2, TGF-ß and 18S (TaqMan) were obtained from Applied Biosciences (Carlsbad, CA), while primers for OPN, IL11, CXCR4, MMP9 and 18S (SYBR) were purchased from Operon (Huntsville, AL) [21]. The PTHrP immunoradiometric assay was purchased from Diagnostic Systems Labs (Brea, CA). Antibodies for phospho-myosin light chain 2 (Ser19, Ser19/Thr18) and anti-Rabbit IgG were procured from Cell Signaling Technologies (Danvers, MA), while ß-actin and Fibronectin antibodies came from Sigma (St. Louis, MO) and Millipore (Billerica, MA), respectively. The ECL chemiluminescence kit was purchased from Amersham (Piscataway, NJ) while the ABTS substrate kit was produced by Vector Laboratories (Burlingame, CA). The mechanotransduction inhibitors Blebbistatin and Y27632 were both procured from Sigma (St. Louis, MO).

### Methods

#### Synthesis of substrates for *in vitro* studies

Polyurethane (PUR) films and polyacrylamide (PAA) hydrogels were synthesized to provide 2D substrates with elastic moduli ranging from ∼1kPa to >1 GPa for *in vitro* studies of mechanosensitivity. PUR substrates were synthesized by reactive liquid molding of a lysine methyl ester diisocyanate (LDI) quasi-prepolymer, a poly(6-caprolactone-*co*-glycolide) triol, and COSCAT 83 bismuth catalyst using published techniques.[18] Briefly, the quasi-prepolymer was synthesized by charging poly(6-caprolactone) triol (PCL, 300 g mol^−1^) to a flask fitted with a reflux condenser and heated to 60°C in an oil bath. Lysine diisocyanate methyl ester (LDI, Kyowa Hakko) was then charged, the reactor immersed in an oil bath maintained at 90°C, and Coscat 83 was added while stirring under dry argon. The reaction was allowed to proceed for three hours under vacuum at 90°C, at which time the reactor was purged with dry argon and the quasi-prepolymer was poured into a vessel stored at 4°C. Structure was verified by nuclear magnetic resonance spectroscopy (NMR, Bruker, 300 MHz), molecular weight was measured by GPC, and % free NCO was measured by titration.

Polyester triols ranging from 300 to 3000 g mol^−1^ (100–1000 g eq^−1^) were synthesized from a glycerol starter, 70% caprolactone and 30% glycolide monomers, and stannous octoate catalyst as described previously [22]. Briefly, the appropriate amounts of dried glycerol, dried 6-caprolactone, glycolide, and stannous octoate (0.1 wt-%) were mixed in a 100-ml flask and heated under an argon atmosphere with mechanical stirring to 135°C. The mixture was allowed to react for ∼30 h and subsequently removed from the oil bath. NMR was used to verify the structure of the polyester triols, with deuterated dichloromethane (DCM) as a solvent. The hydroxyl (OH) number was measured by titration (Metrohm 798 MPT Titrino) according to ASTM D-4662-93 as described [18]. Substrates were synthesized by mixing an appropriate amount of poly(6-caprolactone-*co*-glycolide) triol with LDI quasi-prepolymer, and COSCAT 83 catalyst (Vertellus) for 20s in a Hauschild SpeedMixer™ DAC 150 FVZ-K vortex mixer (FlackTek, Inc, Landrum, SC). The targeted index (ratio of NCO to OH equivalents times 100) was 105. The resultant mixture was poured into the wells of a tissue culture plate and allowed to cure for 24h at 60°C. To facilitate cell adhesion and ensure that the surface chemistry was constant for all substrates tested, fibronectin (Fn) was adsorbed to the surface of the substrates by incubating them in a 4 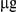/mL solution of Fn in PBS at 4°C overnight.

Polyacrylamide (PAA) hydrogels were synthesized by copolymerizing a 10% solution of acrylamide and bis-acrylamide in water via free-radical polymerization using a redox pair of initiators (tetramethyl ethylene diamine (TEMED) and 10% ammonium persulphate (APS) in water). Additionally, acrylic acid N-hydrosuccinimide (NHS) ester was copolymerized to the surface of the gels. The NHS-acrylate layer was then allowed to react with a solution of Fn in HEPES. To measure the surface concentration of Fn, coated substrates were incubated in a solution of Fn antibody (1∶1000) followed by incubation with a secondary HRP-conjugated antibody. The relative amount of adsorbed antibody was then quantified by reaction with 2′-azino-bis(3-ethylbenzthiazoline-6-sulphonic acid) (ABTS) and subsequent optical density reading at 405nm. All PUR and PAA substrates were prepared at the same surface concentration of Fn that yielded an optical density of 0.12 absorbance units cm^−2^.

#### Dynamic mechanical properties of substrates

Tensile modulus and strength of the PUR films were measured at 37°C for 3×15×1mm films using a TA Instruments Q800 DMA (controlled force displacement ramp, 1 N min^−1^ to 18 N min^−1^) [22]. Storage and loss moduli for the polyacrylamide gels under shear conditions were measured using a TA Instruments AR-G2 Rheometer (TA Instruments, New Castle, DE) at 37°C, using a 20-mm circular head as described previously [15], [23]. Gels were compressed between a heated Peltier plate and a 20-mm upper plate and subjected to an oscillating (0.1–10 Hz) shear strain that was validated to be in the linear range by strain sweep tests.

#### Swelling experiments and calculation of network mesh size

To determine the mesh size of the polymer network, PUR substrates were swollen in dichloromethane for 24h at room temperature, while PAA gels were swollen in water and subsequently lyophilized to determine swelling ratios. The molecular weight between crosslinks, 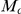, was then determined from the Flory-Rehner equation [24],
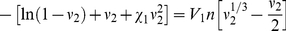
(1)where 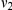 is the volume fraction of the polymer in the swollen mass, 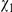 is the Flory-Huggins interaction parameter, 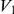 is the molar volume of the solvent, and *n* represents the number of active network chain segments per unit volume. The molecular weight between crosslinks is then given by
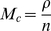
(2)where 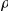 is the density of the polymer network and *n* is the crosslink density (mol cm^−3^). Mesh size, *x_i_*, was then estimated by [25]

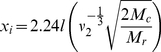
(3)where M_r_ is the molecular weight of the repeat unit and *l* is the carbon-carbon bond length. Assuming the materials were perfectly elastic (Poisson's ratio = 0.5), the elastic and shear moduli (*E*, *G*) are related to the crosslink density:
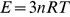
(4)

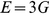
(5)


#### Cell culture

MDA-MB-231 and RWGT2 cells were maintained and cultured at 37°C under 5% CO_2_ in 1× DMEM plus 10% heat inactivated FBS and 1% penicillin/streptomycin. MCF7 cells were maintained and cultured at 37°C under 5% CO_2_ in 1× McCoy's 5A plus 10% heat inactivated FBS, and 1% penicillin/streptomycinm L-glutamine, amino acids, non-essential amino acids and sodium pyruvate each. Cells were harvested with trypsin from substrates after 24h in culture for mRNA extraction (Qiagen RNeasy kit). Conditioned media was collected after 48h in the presence of protease inhibitor for secreted protein analysis. To image cell morphology as a function of substrate rigidity, cells stably expressing GFP were cultured on 0.45kPa (D), 3.3MPa (E) and 1.7GPa (F) substrates and visualized at 24 hours using phase contrast, GFP and DAPI.

#### Quantitative real-time PCR

To measure changes in gene expression, mRNA reverse transcription was carried out using the SuperScript III kit per manufacturer's instructions. Briefly, total RNA was extracted using the RNeasy Mini Kit. The SuperScript III First Strand Synthesis System for quantitative RT-PCR primed with random hexamers was used to synthesize cDNA using between 1 and 5 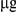 total RNA. The expression of PTHrP, Gli2, and TGF-ß was measured by quantitative RT-PCR using validated TaqMan primers with the 7300 Real-Time PCR System (Applied Biosciences). Assays were performed in triplicate on the RealPlex Machine (Eppendorf) under the following cycling conditions: 95°C for 15 seconds, 58°C for 30 seconds, and 68°C for 30 seconds. Quantification was performed using the absolute quantitative for human cells method using 18S as an internal control. The expression of osteopontin (OPN), interleukin 11 (IL-11), CXCR4, connective tissue growth factor (CTGF), and matrix metalloproteinase- 9 (MMP-9) were determined using SYBR green primers as described previously [21].

#### Immunoradiometric assays

PTHrP protein secretion was measured in conditioned medium using a two-site immunoradiometric assay (IRMA) per manufacturer's instructions. All secreted protein values were normalized for cell number.

#### TGF-ß signaling assay

MDA-MB-231 cells were transiently transfected with 1 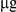 3TPLux, a TGF-ß responsive reporter construct [26], using lipofectamine plus (Invitrogen). pRLTK *Renilla* was cotransfected as a control reporter vector. After 24h in serum-free culture on substrates ranging from 1.7GPa to 0.45kPa, cells were lysed in Passive Lysis Buffer (Promega) and the oxidation of luciferin was measured using a luminometer (TD 20/20, Turner Designs) using a Dual Luciferase Assay Kit (Promega) per manufacturer's instructions. Luciferase activity was then normalized by the *Renilla* control.

#### Western Blotting

Cells cultured as described above were harvested into a radio-immunoprecipitation assay lysis buffer containing a cocktail of protease inhibitors (Roche, Basel, Switzerland). Equal protein concentrations were prepared for loading with NuPAGE sample buffer (Invitrogen) and separated on a gradient (4%–20%) SDS-PAGE gel (Biorad). After transferring to a PVDF, membranes were blocked with 5% BSA in TBS containing 0.1% Tween-20 for 1h at room temperature, followed by incubation with either phospho-myosin light chain 2 (Ser19) or phospho-myosin light chain 2 (Thr18/Ser19) (1∶1000) antibodies overnight at 4°C. After washing, membranes were blotted with anti-rabbit IgG (1∶5000), and bands were detected by enhanced chemiluminescence. Membranes were then stripped and reprobed using an antibody for ß-actin (1∶5000) as a loading control.

#### Inhibition of mechanotransduction

Cells were plated as described above and allowed to adhere for 4h, at which point they were treated with either Y27632 (20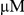) or Blebbistatin (50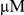). Cells were harvested 24h or 48h post-treatment for mRNA and secreted protein and analyzed as described above. MDA-MB-231, RWGT2 and MCF-7 cells were transfected with either cDNA encoding a dominant active (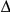
4) or dominant negative (KD
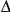
4) mutant of ROCK [27], [28] (a generous gift of Dr. Kazuyuki Itoh, Osaka Medical Center for Cancer and Cardiovascular Diseases, Osaka, Japan) using lipofectamine plus (Invitrogen) per manufacturer's instructions.

#### Inhibition of ROCK in the presence of exogenous TGF-ß

Cells were plated and treated with either Y27632 or Blebbistatin as described previously. Cells were then either co-treated with 5 ng/mL TGF-ß or TGF-ß vehicle (5% BSA-HCl) and harvested for mRNA after 24h for qPCR analysis.

## Results

### 

#### Characterization of PUR and PAA substrates

The characterization of the substrates is summarized in Figure 1. As shown in Figure 1A and B, the PUR films were crosslinked networks. The elastic modulus of the films increased with decreasing equivalent weight, defined as the molecular weight divided by the functionality (f = 3 for a triol). The mesh size of the PUR films ranged from 8.7–24 nm, which is at least 2 orders of magnitude smaller than the size of the cells. Therefore, the cells migrate on the surface since they cannot penetrate the films. The mesh size of the PAA gels was 3.3 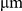, which is also smaller than the size of the cells. As shown in Figure 1C, we obtained reasonable agreement (within a factor of 2) between the measured modulus and that calculated from the swelling experiments using eq (4). PUR substrates were coated with Fn by adsorption, while Fn was grafted to PAA gels (Figure 1D). The concentration of Fn on all surfaces was maintained at 0.12 absorbance units cm^−2^ by controlling the concentration of Fn in the solution.

#### Bone-like mechanical properties stimulate expression of PTHrP

**Figure 2:**
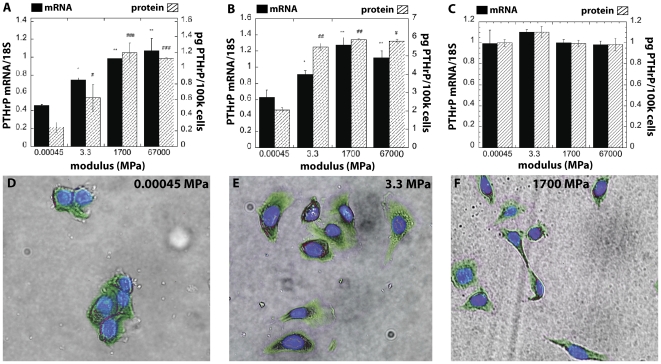
Expression and secretion of PTHrP by osteolytic, metastatic tumor cells increases with increasing substrate modulus. PTHrP mRNA normalized by 18S as measured by qPCR and secreted PTHrP as measured by IRMA for (A) MDA-MB-231 , (B) RWGT2, and (C) MCF-7 cells on substrates of increasing elastic modulus. *,# = p<0.05; **,## = p<0.01; ***,### = p<0.005 compared to 0.00045MPa value. Changes in MCF-7 cells were not significant. (D)–(F) Morphology of MDA-MB-231 cells cultured on substrates of varying elastic modulus 4 hours post-attachment. Nuclei were stained using DAPI (blue) and cells were visualized with both visible light and GFP (green). Similar effects of the modulus on cell morphology were observed for RWGT2 cells, while no effects were observed for MCF-7 cells.

To investigate the effects of substrate modulus on gene expression of cancer cells *in vitro*, we synthesized 2D substrates with tunable elastic moduli ranging from 0.45 kPa to 67 GPa for culture with several cell lines, namely MDA-MB-231 (osteolytic metastatic mammary adenocarcinoma), RWGT2 (osteolytic metastatic lung squamous cell carcinoma), and MCF-7 (non-osteolytic ductal mammary carcinoma). MDA-MB-231 and RWGT2 cells showed 2.5-fold and 2-fold increases in PTHrP mRNA expression respectively in response to substrates with moduli exceeding 1 GPa compared to substrates with moduli <100 kPa (Figure 2A and B). In contrast, MCF-7 cells, which are known not to cause osteolytic lesions showed no difference in PTHrP expression in response to substrate stiffness (Figure 2C). The effects of substrate modulus on PTHrP gene expression followed a sigmoid curve that saturates between 1.7 and 67GPa, which overlaps with the elastic modulus of bone. PTHrP secretion data measured by IRMA changed similarly for all cell lines (Figure 2A–C). Additionally, the morphology of MDA-MB-231 cells changed with substrate rigidity (Figure 2D–F), where GFP (green) is expressed throughout the cell and the DAPI (blue) stain binds to the DNA in the nucleus. Similar effects were observed with RWGT2 cells, and no effects were obtained with MCF7 cells (data not shown).

**Figure 3:**
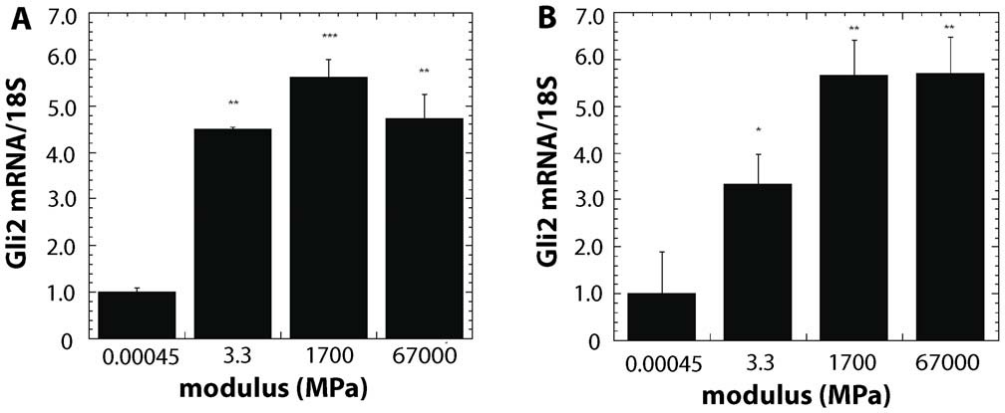
Expression of Gli2 and by MDA-MB-231 and RWGT2 cells increases with increasing substrate modulus. Expression of Gli2 mRNA normalized by 18S as measured by qPCR for (A) MDA-MB-231 and (B) RWGT2 cells on substrates of varying elastic modulus. Gli2 signaling increased 25-fold as the modulus increased from 3.3 to 1700 MPa (data not shown). *,# = p<0.05; **,## = p<0.01; ***,### = p<0.005 compared to 0.00045MPa value.

Expression of Gli2, a transcription factor known to regulate PTHrP [4], also showed a 5-fold increase on hard substrates in both MDA-MB-231 and RWGT2 (Figure 3) but not in MCF-7 cells (data not shown). To verify that these data were not the result of an overall down-regulation of gene expression on soft substrates, several other factors of known importance in bone metastases [29] were examined as summarized in Table 1. Expression of the osteolytic factor IL-11 was relatively insensitive to substrate rigidity for all three cell lines tested. Factors showing the most dramatic change in expression as a function of rigidity include osteopontin (OPN) and MMP-9. Expression of OPN, which is incidentally associated with primary site invasion, was 5–18 times higher when MDA-MB-231 or RWGT2 cells were seeded on soft substrates. Interestingly, expression of OPN by MCF-7 cells was relatively independent of rigidity. Expression of MMP-9 was 10–30 times higher on soft substrates for MDA-MB-231, but the effects of rigidity on MMP-9 expression observed for RWGT2 and MCF-7 cells were substantially smaller.

**Table 1:** Expression of a selected panel of genes associated with osteolytic bone disease as a function of substrate rigidity.

	MDA-MB-231		RWGT2		MCF-7	
Gene	3.3 MPa	1700 MPa	3.3 MPa	1700 MPa	3.3 MPa	1700 MPa
IL-11	1.5	1.4	1.1	1.1	1.1	−1.1
OPN	−18	−18	−5.0	−2.3	1.4	−1.1
MMP-9	−32	−11	−1.3	−1.4	3.7	1.3
CXCR4	−2.0	1.1	1.4	1.7	2.4	1.3
CTGF	1.8	2.0	1.1	1.0	2.4	1.3

Values are listed as fold increase in gene expression compared to that measured for the PAA hydrogel.

#### Substrate-mediated gene expression changes in bone-metastatic cancers are regulated by ROCK-I

**Figure 4:**
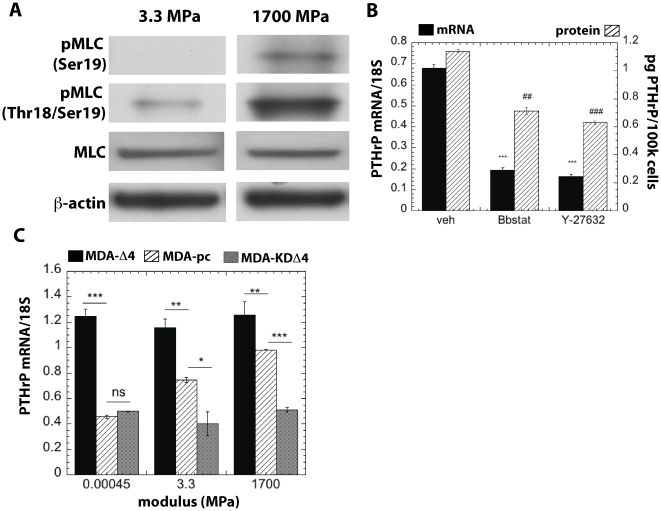
Mechanotransduction signals are regulated by values of the substrate modulus in the MPa range. (A) Western blot analysis of myosin light chain phosphorylation shows higher pMLC expression by MDA-MB-231 cells seeded on more rigid (1700 MPa) relative to softer (3.3 MPa) PUR substrates. (B) Pharmacological inhibition of actomyosin contractility (blebbistatin) and ROCK (Y-27632) decreases PTHrP gene expression and protein secretion in MDA-MB-231 cells. Similar patterns were observed for RWGT2 cells (data not shown). The optimal doses of blebbistatin and Y-27632 were identified to be 50 uM and 20 uM, respectively through dose-response experiments (data not shown). (C) PTHrP is over-expressed and does not increase with rigidity in MDA-MB-231 cells genetically modified to express a constitutively active form of ROCK (MDA-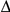
4 cells). Similarly, PTHrP expression does not increase with rigidity in MDA-MB-231 cells genetically modified to express a dominant negative form of ROCK (MDA-KD
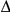
4 cells). MDA cells transfected with a plasmid control (MDA-pc) show significant increases in PTHrP expression with rigidity. *,# = p<0.05; **,## = p<0.01; ***,### = p<0.005.

ROCK signaling regulates actomyosin contractility and cytoskeleton-dependent forces by phosphorylating motor proteins, such as the regulatory MLC, LIMK1/2 and MYPT1 (myosin-binding subunit of MLC) [12]. Thus, ROCK activation leads to increased actomyosin contractility, which led us to question whether the effects of substrate rigidity on PTHrP expression are mediated by ROCK. To determine whether the observed changes in mechanotransduction were linked to expression of osteoclastogenic factors, we first cultured MDA-MB-231 cells on soft (3.3 MPa) and hard (1700 MPa) PUR substrates and measured ROCK activity after 24h by Western blotting. As shown in Figure 4A, cells seeded on more rigid substrates expressed higher levels of phosphorylated MLC (pMLC), implying that ROCK activity increased on more rigid substrates. To further test the hypothesis that modulus effects on PTHrP expression were mediated by ROCK, we pharmacologically inhibited mechanotransduction in MDA-MB-231 and RWGT2 cells on 2 GPa tissue culture polystyrene substrates with blebbistatin or the ROCK inhibitor, Y27632. Inhibition of actomyosin contractility by blebbistatin and inhibition of ROCK-I by Y-27632 induced a significant decrease in PTHrP mRNA expression in both MDA-MB-231 and RWGT2 cells (Figure 4B), suggesting that the upregulation of PTHrP on rigid substrates is mediated by ROCK. Conversely, pharmacologically inhibiting mechanotransduction with either blebbistatin or Y27632 resulted in an increase of OPN mRNA expression in both MDA231 and RWGT2 (data not shown). Neither blebbistatin nor Y27632 had an effect on IL-11 mRNA expression in either cell type (data not shown). MDA-MB-231 cells were also genetically modified to produce constitutively active (MDA-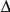
4) and dominant negative (MDA-KD
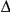
4) forms of ROCK. As shown in Figure 4C, expression of PTHrP by MDA-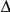
4 cells seeded on 2 GPa substrates was upregulated relative to plasmid control (pc) cells and did not increase with increasing modulus. Expression of PTHrP by MDA-KD
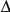
4 cells seeded on the same substrates was down-regulated and did not increase with increasing substrate rigidity. Taken together, the data in Figure 4 indicate that the effects of substrate modulus on PTHrP gene expression are mediated by ROCK.

#### TGF-β mediates the effects of substrate rigidity on Gli2 and PTHrP expression through ROCK

**Figure 5:**
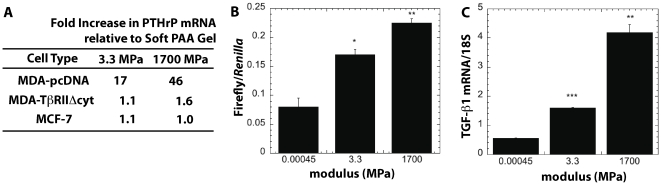
TGF-ß mediates the response of MDA-MB-231 cells to substrate rigidity. (A) PTHrP gene expression increases significantly for MDA-MB-231 cells transfected with the plasmid control (MDA-pc), while MDA-MB-231 cells transfected to express a dominant negative form of the TGF-ß Type II receptor (MDA-TβRII
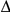
cyt ) and MCF-7 cells that do not express the TGF-ß Type II receptor show no increase in expression with rigidity. (B) TGF-ß signaling was measured in MDA-MB-231 cells transfected with the 3TP-Lux TGF-ß reporter construct. This showed an increase in TGF-ß signaling when cells are grown on rigid substrates. (C) TGF-ß1 mRNA expression by MDA-MB-231 cells increased on more rigid substrates. *,# = p<0.05; **,## = p<0.01; ***,### = p<0.005 compared to 0.00045MPa value.

While Figure 4 shows that ROCK regulates PTHrP expression, TGF-ß is also known to stimulate PTHrP production [5]. Therefore, we investigated its role in regulating the response of the tumor cells to rigidity. As shown in Figure 5A, MDA-MB-231 cells transfected to express a dominant negative form of the TGF-ß Type II receptor (MDA-TβRII
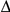
cyt cells) showed only a ≤1.6-fold increase in PTHrP expression as substrate rigidity increased from 0.45 kPa to 1700 MPa. Similarly, MCF-7 cells, which do not express the TGF-ß Type II receptor, also showed ≤1.1-fold increase in PTHrP gene expression with substrate rigidity (Figure 5A). Thus, cells that are non-responsive to TGF-ß did not respond to substrate modulus. Interestingly, intracellular TGF-ß signaling, as measured by the 3TPLux assay, increased with substrate rigidity, as shown in Figure 5B. This is at least in part due to an increase in TGF-ß1 expression by cells on more rigid substrates (Figure 5C).

**Figure 6:**
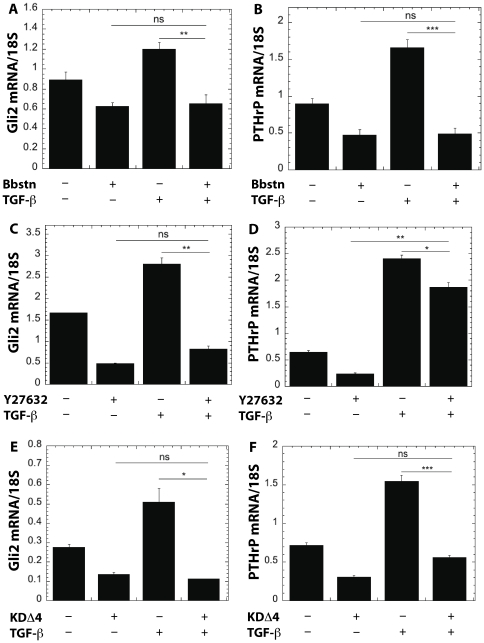
Inhibition of mechanotransduction and ROCK suppresses PTHrP expression induced by exogenous TGF-ß. Treatment with blebbistatin inhibits expression of (A) Gli2 and (B) PTHrP by MDA-MB-231 cells cultured on TCPS. Treatment with 5 ng/ml exogenous TGF-ß increases PTHrP and Gli2 expression, and treatment with both TGF-ß and blebbistatin reduces expression to levels observed for blebbistatin alone. Similarly, treatment of MDA-MB-231 cells with the ROCK inhibitor Y-27632 inhibits (C) Gli2 and (D) PTHrP expression. In the presence of both exogenous TGF-ß and Y-27632, both PTHrP and Gli2 expression are significantly reduced, but PTHrP expression is only partially inhibited relative to treatment with Y-27632 alone. Expression of (E) Gli2 and (F) PTHrP in MDA-MB-231 cells genetically modified to express a dominant negative form of ROCK (MDA-MB-231 KD
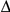
4) is significantly lower than that measured for plasmid control MDA-MB-231 cells. Treatment with exogenous TGF-ß does not significantly increase Gli2 or PTHrP expression in KD
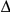
4 cells. *,# = p<0.05; **,## = p<0.01; ***,### = p<0.005.

We next investigated whether blocking ROCK inhibits the effects of exogenous TGF-ß on the stimulation of Gli2 and PTHrP expression by treating cells with TGF-ß and inhibiting mechanotransduction with blebbistatin (Figure 6A and B) or Y27632 (Figure 6C and D). We found that blebbistatin blocked the ability of TGF-ß to stimulate Gli2 and PTHrP expression. Similar results were seen with Y27632 treatments. Using a molecular approach, we treated the KDΔ4 cells with TGF-ß and found that TGF-ß could no longer stimulate Gli2 (Figure 6E) or PTHrP (Figure 6F), thus suggesting that ROCK is required for TGF-ß to stimulate Gli2 and PTHrP.

## Discussion

How tumor cells respond to matrix rigidity is an area of increasing interest, with several groups investigating how the increasing rigidity of soft tissue can alter tumor cell behavior and gene expression. Mechanically transduced signals can induce phenotypic transformation of cells by altering Rho-dependent actomyosin contractility to regulate cellular outcomes, including motility and invasiveness [30], tissue morphogenesis [31], invadopodia activity [32], and stem cell differentiation [33]. While the invasiveness of cancer cells has been reported to increase with the rigidity of the matrix for soft hydrogels [9], [10], [15], [23], the range of substrate rigidity used to investigate cellular responses was generally <100 kPa. A recent study has shown that single cell populations (SCPs) derived from MDA-MB-231 cells exhibited increased proliferation and migration when seeded on matrices with rigidities corresponding to the native rigidities of the organs where metastasis was observed [30]. Thus SCPs targeted specifically to bone proliferated faster and were more invasive on rigid tissue culture polystyrene compared to soft PAA gels. However, whether the differential rigidity of bone alters expression of osteolytic factors by tumor cells has not been investigated. In this study, we show that not only do cells respond with changed behavior when exposed to substrates with rigidities comparable to bone, but also that specific genes involved in tumor-induced bone disease are upregulated. This is especially important in metastatic cancer, since many cancers (such as those of the breast and lung) preferentially metastasize to bone.

Expression of the osteolytic factor PTHrP is more prevalent in bone versus soft tissue metastases as observed in clinical populations [2], [3]. We hypothesized that the differential rigidity of the bone microenvironment induces tumor cells to increase expression of osteolytic factors by altering Rho-dependent actomyosin contractility. To test our hypothesis, we cultured tumor cells on 2D substrates with rigidities ranging from that of breast tissue to mineralized bone. Considering that tumor cell interactions with Fn are important for the development of secondary tumors inside the bone marrow stroma [34], substrates were uniformly coated with Fn to better simulate the bone microenvironment and control the surface chemistry. When MDA-MB-231 or RWGT2 tumor cells were grown on rigid substrates (1.7 and 67 GPa) with moduli bracketing that of mineralized bone matrix (18.4 GPa) [35], both Gli2 and PTHrP expression were 2–4 times higher compared to soft PAA gels (0.45 kPa). Gli2 and PTHrP expression did not increase for moduli above 1 GPa. When cells were seeded on a soft PUR substrate with modulus (3.3 MPa) approaching that of the basement membrane [36], PTHrP expression was intermediate between the soft PAA and the rigid PUR substrates. Similar observations have been reported for MC3T3-E1 pre-osteoblastic cells, which showed higher expression of markers of osteoblast differentiation when cultured on rigid TCPS (2 GPa) compared to 424 and 14 kPa hydrogels [16]. A recent review has reported that actomyosin appeared diffuse for cells cultured on soft gels, in contrast to the stress fibers and strong focal adhesions that predominate when cells were cultured on rigid (e.g., 30–100 kPa) gels or glass (67 GPa) [37]. Based on these observations, it has been suggested that cells cultured on substrates having a modulus equal to or greater than that of stiff gels are in a state of isometric contraction. However, our data show that tumor cells respond to substrate rigidity in the MPa range. While other genes may also increase in addition to Gli2 and PTHrP, we found minimal changes in many of the genes identified previously to be associated with metastasis to bone [29]. Two genes that did change were OPN and MMP-9, which were expressed at a greater amount on softer substrates. This is consistent with clinical data correlating high expression of OPN in metastatic tumor cells [38].

In addition to its role as the factor responsible for humoral hypercalcemia of malignancy (HHM), PTHrP is also expressed by a variety of normal fetal and adult cells, including keratinocytes, mammary epithelial cells, renal tubular epithelial cells, chondrocytes, osteoblasts, and smooth muscle cells [6]. Mechanical stretch induces PTHrP gene expression and protein production in a variety of smooth muscle beds [6], including the abdominal aorta [7], uterus [39], [40], and bladder [8]. In addition, mechanical distension of breast tissue caused by suckling induces a rapid and transient response in PTHrP mRNA expression and protein concentration in lactating mammary tissue in rats [41]. While mechanically induced PTHrP signaling has been shown for normal cells, these effects have not been previously reported for metastatic tumor cells.

When cells encounter a mechanically rigid matrix, integrins become activated, which stimulates RhoGTPas-dependent actomyosin contractility [11]. Thus cells exert actomyosin contractility and cytoskeleton-dependent forces in response to matrix rigidity cues. Inhibition of RhoGTPase signaling in tumor cells by treating with ROCK or myosin 2 inhibitors reduces tumor cell contractility and spreading [11], as well as invadopodia-associated extracellular matrix degradation [15]. Similarly, in this study we observed that expression of phosphorylated myosin light chain (pMLC), which is regulated by ROCK, is increased on rigid substrates. Furthermore, inhibition of tumor cell actomyosin contractility in both pharmacological and genetic models decreased Gli2 and PTHrP expression. A recent study has suggested that increased ROCK signaling contributes to breast cancer metastasis [12]. ROCK expression is increased in metastatic human mammary tumors and breast cancer cell lines, and inhibition of ROCK signaling reduces tumor cell metastasis to bone *in vivo*. Taken together, these observations suggest that inhibiting ROCK or the pathway it stimulates may be an effective approach for treatment of breast cancer metastases in the clinic. Since ROCK is ubiquitously expressed in many tissues and is required for normal mechanotransduction responses in cells, global inhibition in patients may not be an ideal clinical approach. However, the ROCK inhibitors fasudil and Y-27632 have been used successfully in preclinical models of pulmonary and cardiac disease, and a few clinical studies have shown that fasudil is a safe and effective treatment for patients with severe pulmonary hypertension [42].

Mechanical signals induced by substrate rigidity regulate the expression of Gli2 and PTHrP though ROCK. Our observation that the expression of Gli2 by tumor cells is regulated by matrix rigidity is consistent with previous studies suggesting that Hh genes are regulated during development by mechanically transduced signals [43], [44]. While ROCK is ubiquitously expressed in many tissues and cells in the body, the postnatal expression of Gli2 is primarily restricted to the growth plate [45] and hair follicle [46], [47]. Thus Gli2 may be a potentially more useful target than ROCK for treating bone metastases in the clinic. Preclinical studies have indicated that inhibiting Gli2 activity reduces PTHrP expression and osteolysis [48].

Interestingly, substrate rigidity activates ROCK through a TGF-ß-dependent mechanism. As shown in Figure 6, blocking ROCK either pharmacologically or genetically inhibits most of the incremental PTHrP expression resulting from treatment with exogenous TGF-ß. In addition, TGF-ß signaling increases with substrate rigidity, which has also been reported for myofibroblasts cultured on relatively rigid (>10 kPa) hydrogels, where cell-generated forces deform the TGF-ß latent complex resulting in release of soluble TGF-ß [49], [50]. However, in the present study, the expression of TGF-ß1 (but not ß2 or ß3) also increased with rigidity, suggesting a pivotal role for autocrine TGF-ß signaling in this response. This observation is in agreement with previous studies reporting that mechanical stretch or laminar shear stress increases TGF-ß expression and production in endothelial [51], [52] and activated hepatic stellate (HSCs) cells [53]. A mechanotransduction pathway requiring autocrine TGF-ß signaling was found to regulate expression of perlecan in endothelial cells [51]. In another study, transfection of HSCs with a dominant negative form of Rho inhibited the increased production of TGF-ß induced by mechanical stretch [53]. In the bone microenvironment, as osteoclasts resorb host bone, TGF-ß released from the bone matrix stimulates the tumor cells to produce more PTHrP, leading to the vicious cycle of osteoclast-mediated bone resorption associated with metastatic bone disease [5]. Our data show that the increased PTHrP expression induced by exogenous TGF-ß *in vitro* is mediated by the rigidity of the substrate on which the tumor cells are cultured. These observations suggest a role for the differential rigidity of the mineralized bone microenvironment in both the initiation and maintenance of the vicious cycle when osteolytic tumor cells metastasize to bone.

While the interpretation of these experiments is somewhat limited by the use of 2D surfaces, which are known to regulate migration and invasion of tumor cells in ways that differ from 3D matrices [14], the 2D substrates enabled us to independently investigate how rigidity influences gene expression over the 0.45 kPa to 67 GPa range. A recent study has shown that MDA-MB-231 single cell clonal populations proliferated faster on 2D substrates having mechanical properties comparable to that of the organs where metastasis was observed [30]. Thus the response to rigidity in various SCPs in 2D cell culture correlated with the tissue tropism observed *in vivo*. In the present study, tumor cells changed their gene expression patterns when encountering substrates having rigidities in the MPa to GPa range, suggesting that the differential rigidity of the bone microenvironment may contribute to the initial establishment and function of tumor cells in bone. Although the modulus of the tissue that the cells are interacting with in the bone microenvironment is not precisely known, mineralized bone tissue is orders of magnitude more rigid than the primary site [54], and well within the range of the rigid substrates used in this study. We are currently developing a 3D co-culture system *in vivo* to capture more representative features of the bone microenvironment, such as cellular migration and invasion in a 3D matrix and tumor-stromal cell interactions.

In this study, we have shown that tumor cells respond to 2D substrates with rigidities comparable to that of bone by increasing expression and secretion of the osteolytic factor PTHrP. The cellular response is regulated by Rho-dependent actomyosin contractility mediated by TGF-ß signaling. Inhibition of ROCK using both pharmacological and genetic models decreased PTHrP expression. Furthermore, cells expressing a dominant negative form of the TGF-ß receptor did not respond to substrate rigidity, and inhibition of ROCK decreased PTHrP expression induced by exogenous TGF-ß. These observations suggest a role for the differential rigidity of the mineralized bone microenvironment in early stages of tumor-induced osteolysis, which is especially important in metastatic cancer since many cancers (such as those of the breast and lung) preferentially metastasize to bone.

## References

[1] Guise T, Yin J, Taylor S, Kumagai Y, Dallas M (1996). Evidence for a causal role of parathyroid hormone-related protein in the pathogenesis of human breast cancer-mediated osteolysis.. J Clin Invest.

[2] Southby J, Kissin MW, Danks JA, Hayman JA, Moseley JM (1990). Immunohistochemical localization of parathyroid hormone-related protein in human breast cancer.. Cancer Res.

[3] Powell GJ, Southby J, Danks JA, Stillwell RG, Hayman JA (1991). Localization of parathyroid hormone-related protein in breast cancer metastases: increased incidence in bone compared with other sites.. Cancer Res.

[4] Sterling JA, Oyajobi BA (2006). The hedgehog signaling molecule Gli2 induces parathyroid hormone-related peptide expression and osteolysis in metastatic human breast cancer cells.. Cancer Res.

[5] Yin JJ, Selander K, Chirgwin JM, Dallas M, Grubbs BG (1999). TGF-beta signaling blockade inhibits PTHrP secretion by breast cancer cells and bone metastases development.. J Clin Invest.

[6] Philbrick WM, Wysolmerski JJ, Galbraith S, Holt E, Orloff JJ (1996). Defining the roles of parathyroid hormone-related protein in normal physiology.. Physiol Rev.

[7] Pirola CJ, Wang HM, Strgacich MI, Kamyar A, Cercek B (1994). Mechanical stimuli induce vascular parathyroid hormone-related protein gene expression in vivo and in vitro.. Endocrinology.

[8] Yamamoto M, Harm SC, Grasser WA, Thiede MA (1992). Parathyroid hormone-related protein in the rat urinary bladder: a smooth muscle relaxant produced locally in response to mechanical stretch.. Proc Natl Acad Sci U S A.

[9] Paszek MJ, Zahir N, Johnson KR, Lakins JN, Rozenberg GI (2005). Tensional homeostasis and the malignant phenotype.. Cancer Cell.

[10] Paszek MJ, Weaver VM (2004). The tension mounts: mechanics meets morphogenesis and malignancy.. J Mammary Gland Biol Neoplasia.

[11] Butcher DT, Alliston T, Weaver VM (2009). A tense situation: forcing tumour progression.. Nat Rev Cancer.

[12] Liu S, Goldstein RH, Scepansky EM, Rosenblatt M (2009). Inhibition of rho-associated kinase signaling prevents breast cancer metastasis to human bone.. Cancer Res.

[13] Zaman MH, Kamm RD, Matsudaira P, Lauffenburger DA (2005). Computational Model for Cell Migration in Three-Dimensional Matrices.. Biophys J.

[14] Zaman MH, Trapani LM, Sieminski A, MacKellar D, Gong H (2006). Migration of tumor cells in 3D matrices is governed by matrix stiffness along with cell-matrix adhesion and proteolysis.. PNAS.

[15] Alexander NR, Branch KM, Iwueke IC, Guelcher SA, Weaver AM (2008). Extracellular matrix rigidity promotes invadopodia activity.. Curr Biol.

[16] Khatiwala CB, Peyton SR, Metzke M, Putnam AJ (2006). The Regulation of Osteogenesis by ECM Rigidity in MC3T3-E1 Cells Requires MAPK Activation.. J Cellular Physiology.

[17] Engler AJ, Sen S, Sweeney HL, Discher DE (2006). Matrix Elasticity Directs Stem Cell Lineage Specification.. Cell.

[18] Guelcher SA, Dumas J, Srinivasan A, Didier JE, Hollinger JO (2008). Synthesis, mechanical properties, biocompatibility, and biodegradation of polyurethane networks from lysine polyisocyanates.. Biomaterials.

[19] Guelcher S (2008). Biodegradable polyurethanes: synthesis and applications in regenerative medicine.. Tissue Eng.

[20] Guise TA, Yoneda T, Yates AJ, Mundy GR (1993). The combined effect of tumor-produced parathyroid hormone-related protein and transforming growth factor-alpha enhance hypercalcemia in vivo and bone resorption in vitro.. J Clin Endocrinol Metab.

[21] Javelaud D, Mohammad KS, McKenna CR, Fournier P, Luciani F (2007). Stable overexpression of Smad7 in human melanoma cells impairs bone metastasis.. Cancer Res.

[22] Hafeman A, Li B, Yoshii T, Zienkiewicz K, Davidson J (2008). Injectable biodegradable polyurethane scaffolds with release of platelet-derived growth factor for tissue repair and regeneration.. Pharm Res.

[23] Enderling H, Alexander NR, Clark E, Branch KM, Estrada L (2008). Dependence of invadopodia function on collagen fiber spacing and crosslinking: computational modeling and experimental evidence.. Biophys J.

[24] Sperling LH (2001). Introduction to Physical Polymer Science.

[25] Raeber GP, Lutolf MP, Hubbell JA (2005). Molecularly engineered PEG hydrogels: a novel model system for proteolytically mediated cell migration.. Biophys J.

[26] Kakonen SM, Selander KS, Chirgwin JM, Yin JJ, Burns S (2002). Transforming growth factor-beta stimulates parathyroid hormone-related protein and osteolytic metastases via Smad and mitogen-activated protein kinase signaling pathways.. J Biol Chem.

[27] Narumiya S, Tanji M, Ishizaki T (2009). Rho signaling, ROCK and mDia1, in transformation, metastasis and invasion.. Cancer Metastasis Rev.

[28] Itoh K, Yoshioka K, Akedo H, Uehata M, Ishizaki T (1999). An essential part for Rho-associated kinase in the transcellular invasion of tumor cells.. Nat Med.

[29] Kang Y, Siegel PM, Shu W, Drobnjak M, Kakonen SM (2003). A multigenic program mediating breast cancer metastasis to bone.. Cancer Cell.

[30] Kostic A, Lynch CD, Sheetz MP (2009). Differential matrix rigidity response in breast cancer cell lines correlates with the tissue tropism.. PLoS One.

[31] Paszek MJ, Weaver VM (2004). The tension mounts: mechanics meets morphogenesis and malignancy.. J Mammary Gland Biol Neoplasia.

[32] Alexander NR, Branch KM, Parekh A, Clark ES, Iwueke IC (2008). Extracellular Matrix Rigidity Promotes Invadopodia Activity.. Curr Biol.

[33] Engler AJ, Sen S, Sweeney HL, Discher DE (2006). Matrix elasticity directs stem cell lineage specification.. Cell.

[34] Van der Velde-Zimmermann D, Verdaasdonk MA, Rademakers LH, De Weger RA, Van den Tweel JG (1997). Fibronectin distribution in human bone marrow stroma: matrix assembly and tumor cell adhesion via alpha5 beta1 integrin.. Exp Cell Res.

[35] Cuppone M, Seedhom BB, Berry E, Ostell AE (2004). The longitudinal Young's modulus of cortical bone in the midshaft of human femur and its correlation with CT scanning data.. Calcif Tissue Int.

[36] Candiello J, Balasubramani M, Schreiber EM, Cole GJ, Mayer U (2007). Biomechanical properties of native basement membranes.. FEBS Journal.

[37] Discher DE, Janmey P, Wang YL (2005). Tissue cells feel and respond to the stiffness of their substrate.. Science.

[38] Hotte SJ, Winquist EW, Stitt L, Wilson SM, Chambers AF (2002). Plasma osteopontin: associations with survival and metastasis to bone in men with hormone-refractory prostate carcinoma.. Cancer.

[39] Daifotis AG, Weir EC, Dreyer BE, Broadus AE (1992). Stretch-induced parathyroid hormone-related peptide gene expression in the rat uterus.. J Biol Chem.

[40] Thiede MA, Daifotis AG, Weir EC, Brines ML, Burtis WJ (1990). Intrauterine occupancy controls expression of the parathyroid hormone-related peptide gene in preterm rat myometrium.. Proc Natl Acad Sci U S A.

[41] Thiede MA, Rodan GA (1988). Expression of a calcium-mobilizing parathyroid hormone-like peptide in lactating mammary tissue.. Science.

[42] Barman SA, Zhu S, White RE (2009). RhoA/Rho-kinase signaling: a therapeutic target in pulmonary hypertension.. Vasc Health Risk Manag.

[43] Nowlan NC, Prendergast PJ, Murphy P (2008). Identification of mechanosensitive genes during embryonic bone formation.. Plos Computational Biology.

[44] Tang GH, Rabie AB, Hagg U (2004). Indian hedgehog: a mechanotransduction mediator in condylar cartilage.. J Dent Res.

[45] Miao D, Liu H, Plut P, Niu M, Huo R (2004). Impaired endochondral bone development and osteopenia in Gli2-deficient mice.. Exp Cell Res.

[46] Mill P, Mo R, Fu H, Grachtchouk M, Kim PC (2003). Sonic hedgehog-dependent activation of Gli2 is essential for embryonic hair follicle development.. Genes Dev.

[47] Eichberger T, Kaser A, Pixner C, Schmid C, Klingler S (2008). GLI2-specific transcriptional activation of the bone morphogenetic protein/activin antagonist follistatin in human epidermal cells.. J Biol Chem.

[48] Johnson RW, Nguyen MP, Padalecki SS, Grubbs BG, Merkel AR (Submitted) TGF-β promotion of Gli2 induced PTHrP expression is independent of canonical Hedgehog signaling.. Cancer Res.

[49] Wipff PJ, Rifkin DB, Meister JJ, Hinz B (2007). Myofibroblast contraction activates latent TGF-1 from the extracellular matrix.. J Cell Biol.

[50] Wells RG, Discher DE (2008). Matrix elasticity, cytoskeletal tension, and TGF-beta: the insoluble and soluble meet.. Science Signaling.

[51] Baker AB, Ettenson DS, Jonas M, Nugent MA, Iozzo RV (2008). Endothelial cells provide feedback control for vascular remodeling through a mechanosensitive autocrine TGF-beta signaling pathway.. Circ Res.

[52] Ohno M, Cooke JP, Dzau VJ, Gibbons GH (1995). Fluid shear stress induces endothelial transforming growth factor beta-1 transcription and production. Modulation by potassium channel blockade.. J Clin Invest.

[53] Sakata R, Ueno T, Nakamura T, Ueno H, Sata M (2004). Mechanical stretch induces TGF-beta synthesis in hepatic stellate cells.. Eur J Clin Invest.

[54] Moore SW, Roca-Cusachs P, Sheetz MP (2010). Stretchy proteins on stretchy substrates: the important elements of integrin-mediated rigidity sensing.. Dev Cell.

